# A new validated score for detecting patient-reported success on postoperative ICIQ-SF: a novel two-stage analysis from two large RCT cohorts

**DOI:** 10.1007/s00192-016-3070-0

**Published:** 2016-07-05

**Authors:** Debjyoti Karmakar, Alyaa Mostafa, Mohamed Abdel-Fattah

**Affiliations:** 1 Department of Obstetrics and Gynaecology, University of Aberdeen, Aberdeen, UK; 2 Department of Urogynaecology, Mercy Hospital for Women, Heidelberg, Melbourne, Victoria 3084 Australia

**Keywords:** Tension free vaginal tapes, Stress urinary incontinence, Patient Global Impression of Improvement (PGI-I), International Consultation of Incontinence Questionnaire-Short Form (ICIQ-SF), Patient Reported Outcome Measures (PROM)

## Abstract

**Introduction and hypothesis:**

The Patient Global Impression of Improvement (PGI-I) and International Consultation of Incontinence Questionnaire – Short Form (ICIQ-SF) are validated instruments for the assessment of patient reported outcome measures (PROM) following treatment of stress urinary incontinence (SUI). However, there is a paucity of evidence as to what represents a successful postintervention ICIQ-SF score. To determine the correlation between the postoperative ICIQ-SF scores with the PGI-I outcomes, the latter was considered one of the standard PROM following surgical treatment for SUI. The aim of this study was to determine, and if appropriate validate, an ICIQ-SF cut-off score that can predict a successful PROM as determined by PGI-I.

**Methods:**

Four large datasets yielding 674 ICIQ-SF score/PGI-I outcome data pairs were used in this study for (a) determining and (b) validating the cut-off ICIQ-SF score for a successful PGI-I outcome. Two long-term follow-up datasets were used representing follow-up periods of 3 and 8 years of a randomized controlled trial (RCT) performed between April 2005 and April 2007 in a tertiary urogynaecology centre in Scotland, UK. All patients had urodynamic SUI or mixed urinary incontinence (MUI, with predominant SUI) and were randomized to treatment with either an inside-out or an outside-in transobturator tape (TVT-O or TOT, respectively) as a sole procedure. The datasets yielded 432 ICIQ-SF score/PGI-I outcome data pairs. Successful outcome was defined as “very much improved/much improved” on the PGI-I scale. SPSS v. 22.0 (IBM Corp., Armonk, NY) was used for all statistical analyses. The correlations and cut-off scores generated were then validated on two independent datasets representing the 1-year and 4-year follow-up periods of the multicentre RCT in six units in the UK. The datasets yielded 242 ICIQ-SF score/PGI-I outcome data pairs. All patients had urodynamic SUI or MUI (with predominant SUI) and were randomized to either adjustable single incision minisling (SIMS) or TVT-O.

**Results:**

Significant correlations at the 0.01 level (two-tailed) were clearly demonstrated between ICIQ-SF scores at follow up and PGI-I outcomes in terms of success/failure in both the generation and validation datasets. Higher ICIQ-SF scores correlated with a ‘poorer’ PGI-I score. Using ROC analysis, a postoperative ICIQ-SF score of 6 was validated as approximately 90 % sensitive and 85 % specific for success/failure with a high Cohen’s kappa coefficient of 0.83 (95 % CI 0.74 – 0.89).

**Conclusions:**

This two-stage study provided a robust well-validated postoperative ICIQ-SF cut-off score (of 6/21) that is likely to be associated with a patient-reported successful outcome on the PGI-I following surgical treatment with a midurethral sling in women at different stages of follow-up over 1 – 8 years. Such a cut-off score could enable the comparison of results between various studies and serve as a valuable guide for surgeons to counsel patients before and/or after surgical treatment. Our study fills a research gap in providing a way to compare trial results when baseline ICIQ-SF scores are not available.

## Introduction

Urinary incontinence (UI) is a distressing condition that negatively affects women’s quality of life (QoL). Therefore, assessment and evaluation of patients’ symptom severity and QoL prior to and after an intervention are essential [1, 2]. A number of self-assessment questionnaires have been recommended [3, 4], but simplicity and brevity are important features in designing patient self-assessment questionnaires [3, 4]. The International Consultation of Incontinence Questionnaire – Short Form (ICIQ-SF) is a validated subjective measure of severity and impact of UI on the QoL in women [1]. The ICIQ-SF is formed of six items of which four main items ask for rating of UI symptoms in the past 4 weeks. The scores for items 3, 4 and 5 are taken for the final ICIQ-SF score. Items 1 and 2 are demographic and the final item is a self-diagnostic item for the type of UI. The Patient Global Impression of Improvement (PGI-I) is a seven-point scale instrument of patient reported outcome measures (PROM) which is validated to assess PROM following treatment of stress UI (SUI) [2]. However, there is a paucity of evidence as to what represents a successful postintervention ICIQ-SF score, and this is further complicated when the baseline score is not available [5]. There is a significant drive to conduct and compare the long-term follow-up results of randomized controlled trials (RCTs) related to UI interventions; however, some of these RCTs do not have baseline ICIQ-SF scores simply because the ICIQ-SF was developed only in 2004.

The objective of this study was to determine the correlation between the postoperative ICIQ-SF scores and the PGI-I outcome. The latter is considered the standard PROM following surgical treatment for SUI. If we did indeed find a significant correlation, we intended to determine, and if appropriate validate, a postoperative ICIQ-SF cut-off score that could predict a successful PROM as determined by PGI-I. Such a score would facilitate the comparison of results among clinical trials using different tools to asses PROM and especially those in which long-term follow-up is feasible. Such a score could also be a valuable aid in the counselling of patients and facilitating their postoperative follow-up.

## Materials and methods

Two PROM assessment tools were assessed: ICIQ-SF and PGI-I. ICIQ-SF has a maximum score of 21; the higher the score, the more severe is the UI, but there is no normal score (Appendix 1). The PGI-I is a seven-point transition scale that comprises a single question asking patients to rate their urinary tract condition now as compared with how it was before treatment on a scale from 1 (very much better) to 7 (very much worse). Outcomes of “much improved” or “very much improved” are globally accepted as successful outcomes, and were therefore used in this study to indicate a successful surgical outcome.

### Methodology for assessing the correlation between the two instruments and generation of ICIQ-SF cut-off score for success

We used two long-term follow-up datasets (3 and 8 years) from a RCT (the E-TOT study; Table 1) performed between April 2005 and April 2007 with follow-up until 2015 in a tertiary urogynaecology centre in Scotland, UK [6, 7]. All patients had urodynamic SUI or mixed UI (MUI, with predominant SUI) and were randomized to treatment with either inside-out TVT-O (Ethicon Inc., Somerville, NJ) or Aris outside-in TOT (Coloplast, Peterborough, UK) as a sole procedure. The datasets yielded 432 complete ICIQ-SF score/PGI-I outcome data pairs. These datasets were from patients who responded to follow-up postal questionnaire packs which also included symptom severity, QoL and sexual function questionnaires. We used the usual convention for the PGI-I transition scale from 1 (very much better) to 7 (very much worse). We determined the correlation between absolute scores. We did not stratify the ICIQ-SF scores into mild, moderate and severe, as this procedure has been shown to have limited validity [9].

**Table 1:** Datasets

Dataset	Study	Follow-up (years)	Number of data pairs	Total
Correlation and generation of cut-off score	E-TOT RCT	3	238	432
		8	194	
Validation	SIMS RCT	1	131	242
		4	111	

### Methodology for the validation of the novel ISIC-SF cut-off score for success

The correlations and cut-off scores generated were validated on an independent dataset representing the 1-year and 4-year follow-up from another multicentre RCT (the pilot SIMS study; Table 1) for surgical management of SUI in women [8] (Mostafa et al. Submitted for publication). Similar to the E-TOT RCT, all patients had urodynamic SUI or MUI (with predominant SUI) and were randomized to treatment with either adjustable anchored SIMS (Ajust; C. R. Bard Inc., NJ) or TVT-O, (Ethicon Inc., Somerville, NJ) as a sole procedure. These patients were respondents to the same follow-up postal questionnaire pack as in the E-TOT RCT. The datasets yielded 242 ICIQ-SF score/PGI-I outcome data pairs.

SPSS v. 22.0 (IBM Corp., Armonk, NY) was use for all statistical analyses. One-way ANOVA was used to determine whether the mean posttreatment ICIQ-SF scores were significantly different between the different PGI-I categories to eliminate confounders. Correlation analysis was then done yielding Pearson coefficients. Receiver operator characteristic (ROC) curves were generated for each cohort with ICIQ-SF scores and PGI-I outcomes (success/failure) as variables using the SPSS ROC function. These were used to generate sensitivity and specificity cut-off values. Tukey type post-hoc analysis was performed.

## Results

### ICIQ-SF cut-off score for success

The correlation between the ICIQ-SF score at follow-up and PGI-I outcome was highly significant at the 0.01 level (two-tailed; Pearson coefficient −0.629). Higher ICIQ-SF scores correlated with a ‘poorer’ PGI-I score. The ROC analysis and the analysis of the sensitivities and specificities in both datasets gave an ICIQ-SF cut-off score of 6 as approximately 90 % sensitive and 85 % specific for success or failure as judged by the PGI-I, This was deemed to be both clinically and statistically sufficiently robust to proceed to the validation stage (Table 2).

**Table 2:** Consolidated specificities and sensitivities generated by ROC analysis of ICIQ-SF scores at follow-up and success/failure on PGI-I

Dataset	ICIQ-SF score	Sensitivity	1 − specificity
Generation dataset	4.500	0.982	0.348
	5.500	0.919	0.168
	6.500	0.854	0.152
	7.500	0.688	0.115
Validation dataset	4.500	0.778	0.060
	5.500	0.823	0.120
	6.500	0.889	0.158
	7.500	0.903	0.328

### Validation of the generated ICIQ-SF cut-off score

As stated above, the correlation between the ICIQ-SF score at follow-up and PGI-I outcome was significant at the 0.01 level (two-tailed; Pearson coefficient: −0.630). The ROC curve analysis evaluating the relationship between ICIQ-SF score and PGI-I success/failure outcome gave an ICIQ-SF cut-off score of 6 for use as a test for success in the validation cohort and compared with actual outcomes. Cohen’s kappa coefficient for the correlation between the two datasets was 0.83 (95 % CI 0.74 – 0.89), clearly indicating the validity of the cut-off score.

## Discussion

Both PGI-I and ICIQ-SF are validated methods in postintervention assessment of UI. Despite being widely used in clinics and research settings and the wealth of information about ICIQ-SF and PGI-I separately, there has been a longstanding and ongoing effort to robustly correlate these two valuable instruments so that results from different authors/research groups can be compared [1–5]. The PGI-I is a global index that is widely used to rate the response of a condition to a therapy (transition scale). It is a simple, direct, easy to use scale that is intuitively understandable by clinicians and patients [3]. The PGI-I has been found to have excellent construct validity when compared with various assessment variables: incontinence episode frequency, the Incontinence Quality of Life Questionnaire, and the fixed volume (400 mL) stress pad test [5]. Such global ratings can be precise when used to assess the same person over time, but they have a degree of imprecision across the spectrum of different people in whom they might be used [5]. PGI-I has recently been found by Hossack and Woo to be a valid assessment tool even following prostatectomy, and PGI-I is now being looked at with great interest in urology research. It has shown excellent correlation with the symptom score and QoL index in urology research [17]. Most recently PGI-I was used in the Bladder Ultrasound Study (BUS trial) funded by the Health Technology Assessment Programme with a follow-up of up to 20 months in women with overactive bladder [16].

The ICIQ-SF is a subjective measure of severity of urinary loss and QoL for those with UI. This PROM tool takes 5 min or less time to administer and no training, and hence is widely used in both clinical and research settings. ICIQ-SF has been tested and validated in men and women with primary SUI. Cut-off scores for severity of UI in women have been reported by Klovning et al. in a cohort of 1,812 women responding to a general health questionnaire [9]. Score ranges were 1 – 5 (slight), 6 – 12 (moderate), 13 – 18 (severe) and 19 – 21 (very severe).

ICIQ-SF has the ability to detect changes after both conservative and surgical treatment. The minimal detectable change (MDC) and minimally clinically important difference (MCID) after intervention for UI are not yet established, but there has been initial investigation in this area [5–10]. Sirls et al. assessed 597 women in the TOMUS RCT and found that the minimum important difference (MID) for the ICIQ-SF in a population of women with stress-predominant UI is −5 for assessment at 12 months and −4 for assessment at 24 months [10]. They concluded that MID may be overestimated in surgical cohorts because of uniformly high preoperative scores without significant variability that show a large improvement after treatment [10]. Nyström et al. also found that the changes in ICIQ-SF and ICIQ-LUTS QoL scores in women with SUI after pelvic floor muscle training reflected clinically relevant improvements [11]. They included 218 women with a 4-month follow-up. The MID was defined as the mean change in score in women who experienced a small improvement. Similar to our results, Nyström et al. [11] found that PGI-I outcome correlated significantly with the ICIQ-SF score (*r* = 0.547, *P* < 0.0001) while the MID was −2.52. The clear discrepancy between MCID in the above studies shows that more research is needed in this area. Nyström et al. hypothesized that PGI-I outcome may be better correlated with postoperative ICIQ-SF score than the change in the score over time which was clearly lower than that found by Sirls et al. [10].

In our study, we analysed the results from two large datasets representing the PROM up to 8 years following treatment with a midurethral sling (MUS). In this RCT which was started in 2005, a baseline ICIQ-SF score was not available. A clear strong correlation between PGI-I outcome and ICIQ-SF score was shown, and we identified a postoperative ICIQ-SF score of 6 as a marker of successful outcome according to PGI-I with reasonable specificity (85 %) and sensitivity (90 %). The score was then validated in two datasets representing follow-up periods of up to 4 years in a separate RCT also assessing outcomes following treatment with a MUS, but where the baseline ICIQ-SF scores were known. This demonstrated that the results are fully validated for the majority of women undergoing treatment with a MUS.

NICE (National Institute for Care and Health Excellence) guidelines on UI (CG171) [12], a Cochrane review [13], and most recently a SCENIHR (Scientific Committee on Emerging and Newly Identified Health Risks) report all encourage authors to undertake RCTs with long-term follow-up and to compare the results with those of relatively old RCT. Many studies, like ours, will not have had the chance to use the ICIQ-SF at baseline given the timeline of trials and the publication of ICIQ-SF in 2004. Our results showing the correlation between the ICIQ-SF score and PGI-I outcome and the validity of the ICIQ-SF cut-off score would enable the ICIQ-SF to be administered postoperatively in these trials and enable their results to be compared with those of other RCTs in a meta-analysis. This would allow the clinical community to fill the gap in the evidence for the long-term outcomes following surgical treatment of SUI. Unlike MCID, our highlighted ICIQ-SF cut-off score was not affected by the length of follow-up up to 8 years. In-addition, this novel validated ICIQ-SF cut-off score will be a valuable aid in counselling patients during their follow-up after surgery for incontinence. A postoperative ICIQ-SF score of ≤6 is likely to translate to a patient-reported successful outcome according to the PGI-I.

Recently, Larsen et al. [14] compared the PGI-I and the ICIQ-SF score in women undergoing surgery for UI or pelvic organ prolapse and found that the PGI-I score correlates better with the postoperative ICIQ score than the change in score after surgery. They concluded that this may be due to patients’ recall bias, and warned that PGI-I tends to overestimate patient-reported success compared with the change in ICIQ score after treatment.

### Methodology

In the accompanying editorial, Cartwright et al. [15] questioned their methodology of converting the individual scores for both questionnaires to the same scale as the numerical values assigned to each category in the underlying items are somewhat arbitrary [15]. The use of four large datasets assessing different follow-up times after treatment with a MUS (1, 3, 4 and 8 years) is a major strength of our study. The fact that the cut-off score did not seem to be affected by the length of follow-up or the type of MUS used is important for both clinical and research use. A potential limitation is that correlating two questionnaires that measure the same clinical intervention may be inappropriate. Cartwright et al. [15] argue that the differences between the PGI-I and the ICIQ reflect true differences in what they measure. PGI-I provides a more global overview of treatment success that is more likely to fully encompass the range of benefits and harms of surgery compared to a disease-specific questionnaire such as ICIQ.

## Conclusions

This two-stage study provided a robust well-validated postoperative ICIQ-SF cut-off score (of 6/21) that is likely to translate to a patient-reported successful outcome on PGI-I following surgical treatment with a MUS in women at different stages of follow-up from 1 to 8 years. Such a cut-off score may enable comparison of results between various studies and serve as a valuable guide for surgeons to counsel patients before and/or after surgical treatment. Our study fills the research gap in providing a way to compare trial results when baseline ICIQ-SF scores are not available.
